# Plasma metabolomic signatures of the no-reflow phenomenon in stroke patients following thrombectomy

**DOI:** 10.3389/fneur.2026.1740882

**Published:** 2026-06-19

**Authors:** Xingtong Li, Xi Chen, Honghua Pan, Junhua Wu, Shuai Mi, Ying Jiang, Linke Zhou, Yu Wang, Min Wu, Xianhua Hou, Zhenhua Zhou

**Affiliations:** 1 Department of Neurology, Southwest Hospital, Third Military Medical University (Army Medical University), Chongqing, China; 2 Guilin Municipal People's Hospital, Guangxi Zhuang Autonomous Region, Guilin, China

**Keywords:** biomarkers, ischemic stroke, LC-HRMS, metabolites, no-reflow phenomenon, thrombectomy

## Abstract

**Background:**

Although successful recanalization of the occluded artery is achieved, no-reflow phenomenon (NRP) becomes a main contributor to poor prognosis in patients with acute ischemic stroke. There are some laboratory results to represent biomarkers of the no-reflow phenomenon. However, few studies have characterized the metabolomic signature of NRP. Using high-performance liquid chromatography–tandem mass spectrometry (LC–MS)-based method, this study aims to characterize the plasma metabolites associated with NRP.

**Methods:**

A total of 34 patients with acute large vessel occlusion in anterior circulation who underwent successful thrombectomy with final angiographic expanded Treatment in Cerebral Infarction score of 2c-3 score were enrolled (19 without NRP and 15 with NRP). Fasting venous blood collected 24 h after the procedure was centrifuged and subjected to metabolomic analysis.

**Results:**

We identified 29 differentially expressed plasma metabolites, the majority of which were phosphatidylcholine (PC) species. Among them, PC(20:4(5Z,8Z,11Z,14Z)/P-16:0) showed the most significant alteration and exhibited robust predictive performance (AUC = 0.846). The most prominently disrupted metabolic pathway was glycerophospholipid metabolism, particularly PC-mediated pathways, which appeared to play a central role in the association with of NRP.

**Conclusion:**

This study depict the plasma metabolic profile of NRP patients following stroke thrombectomy, and discover that phosphatidylcholine-dominated metabolites and related pathways may play a potential role in the occurrence of NRP. These metabolic biomarkers demonstrate promising discriminative ability and may help identify high-risk patients at an early stage, providing new targets for mechanism research and therapeutic intervention.

## Introduction

Globally, acute ischemic stroke (AIS) is the second leading cause of mortality and long-term disability (1, 2). For patients with large vessel occlusion, the current standard of care—intravenous thrombolysis followed by mechanical thrombectomy (3)—achieves high rates of macrovascular recanalization. However, approximately 30–50% of patients with successful recanalization exhibit poor functional outcomes, a clinical paradox largely attributed to the no-reflow phenomenon (NRP) (4). This critical pathological state reflects microcirculatory dysfunction despite restored arterial patency, resulting in incomplete tissue reperfusion and persistent ischemia (5). We recently reported that the poor prognosis attributable to the “no-reflow” persisted even after the successful thrombectomy with inter-arterial thrombolysis; however, the exploration of this phenomenon remains full of challenges (6). Pathophysiologically, NRP is mainly attributed to microvascular obstruction, endothelial swelling, pericyte contraction, leukocyte adhesion, and oxidative stress-induced injury, all of which collectively result in persistent hypoperfusion of ischemic brain (7). Imaging correlates on CT perfusion such as reduced relative cerebral blood flow (rCBF) and relative cerebral blood volume (rCBV), together with prolonged mean transit time (MTT), have been used to identify NRP (8). Moreover, several circulating biomarkers—including inflammatory cytokines and saturation of the fatty acids, have also been proposed to be associated with ischemic stroke (9). However, studies on the metabolic profile of patients with NRP after stroke have not been reported yet.

Currently, NRP is diagnosed primarily with advanced neuroimaging techniques, including CT perfusion (CTP), MRI perfusion-weighted imaging (PWI), and transcranial Doppler (TCD). These methods assess microvascular perfusion by measuring parameters such as cerebral blood flow (CBF), cerebral blood volume (CBV), and mean transit time (MTT) (9). However, these approaches have several limitations: they are costly, time-consuming, require specialized equipment and expertise, expose patients to radiation or contrast agents, and may not be readily available in all clinical settings. Therefore, there is an urgent need for minimally invasive, easily accessible biomarkers that can reflect the pathophysiological processes associated with NRP.

Several circulating biomarkers have been previously reported in association with the no-reflow phenomenon. These include markers of inflammation and coagulation abnormalities, reflecting the complex pathophysiology of microvascular obstruction after successful recanalization (10). However, these conventional biomarkers primarily capture isolated aspects of the pathological cascade and may not fully reflect the global metabolic perturbations underlying NRP.

Metabolomics focus on the global profiling of endogenous small-molecule metabolites (<1 kDa), their dynamic fluctuations, and regulatory networks (11). Since metabolites can pass through the blood–brain barrier and mirror early pathophysiological shifts, they provide a new prospective for exploring the potential mechanism underlying NRP (12). Indeed, metabolomic interrogations have already pinpointed stroke-associated metabolites linked to onset, progression, and long-term outcome, nominating putative diagnostic biomarkers and enabling precision therapeutics (13). Cerebral ischaemia/reperfusion injury (CIRI) precipitates NRP via metabolic perturbations that encompass oxidative stress, calcium overload, and inflammatory cascades; metabolites such as pyruvate and succinate exhibit time-dependent fluctuations during CIRI, underscoring their potential as tractable targets (14–16).

These findings highlight the potential of metabolomics to uncover novel biomarkers and therapeutic targets for NRP. Therefore, the present study employed untargeted liquid-chromatography high-resolution mass spectrometry (LC-HRMS) to delineate differential plasma metabolites and perturbed metabolic pathways, and to evaluate their diagnostic utility for NRP after successful endovascular reperfusion.

## Materials and methods

This study screened 60 participants recruited from the Department of Neurology, First Affiliated Hospital of Army Medical University, between January and October 2024. The study was approved by the Ethics Committee of the First Affiliated Hospital of Army Medical University (Approval (A)KY2024154). According to the exclusion criteria below, 34 patients were finally enrolled, consisting of 15 with no-reflow and 19 with successful reperfusion.

**Figure 1:**
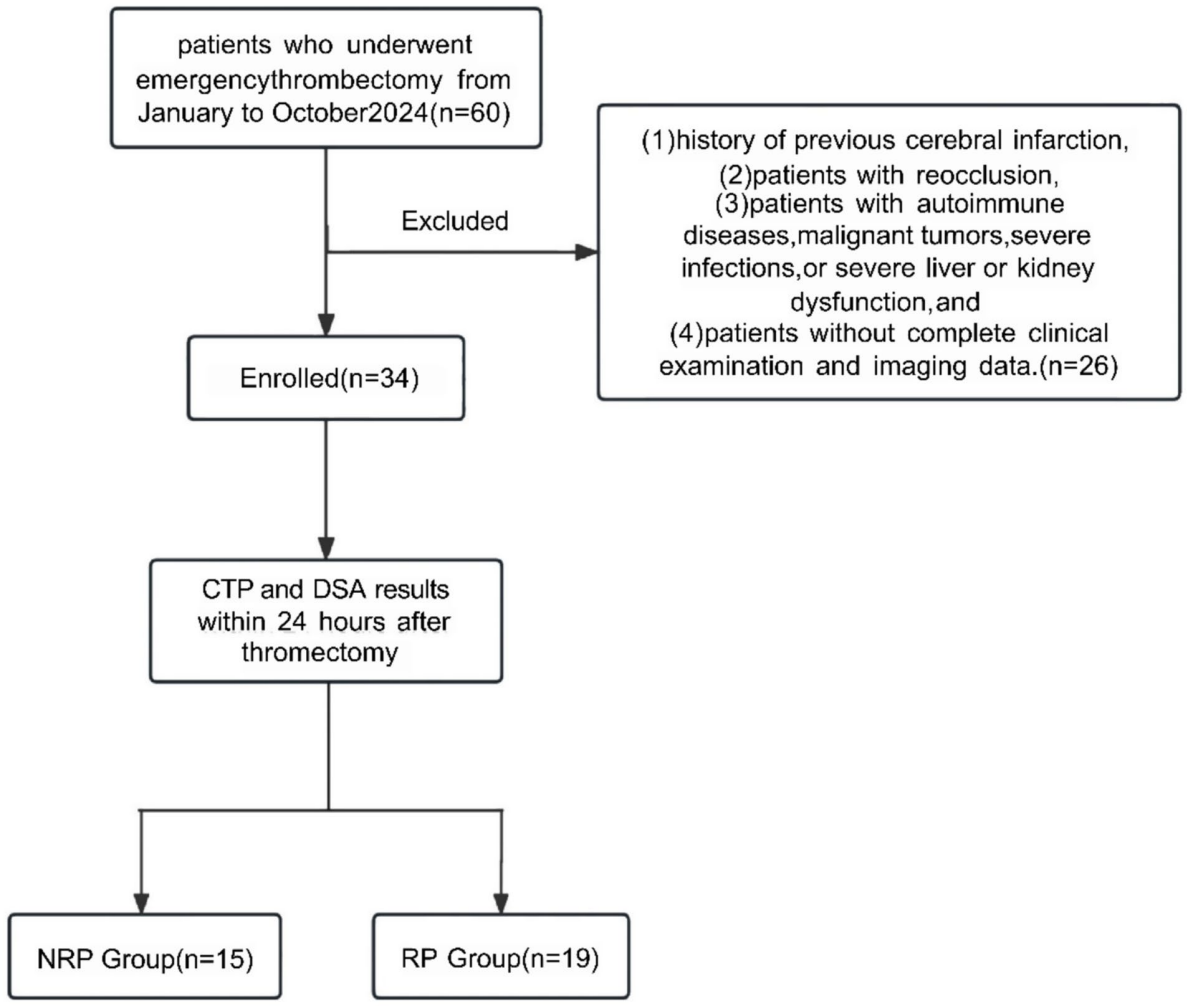
Study workflow. After screening, 34 patients with acute ischemic stroke (AIS) underwent serum metabolomic profiling to identify potential biomarkers for no-reflow phenomenon (NRP) diagnosis.

The inclusion criteria were as follows: (1) Onset time of stroke symptoms ≤ 24 h; (2) Age ≥ 18 years; (3) Clinical diagnosis of acute ischaemic stroke; (4) Imaging confirmation of anterior-circulation large-vessel occlusion; (5) Postoperative angiographic assessment shows expanded Treatment in Cerebral Infarction (eTICI) grade 2c–3; (6) CT perfusion imaging (CTP) performed within 24 h postoperatively. Exclusion criteria included: (1) history of previous cerebral infarction, to avoid confounding effects from pre-existing brain injury on metabolomic profiles, (2) patients with reocclusion, because autoimmune conditions can significantly alter systemic inflammatory and metabolic states, (3) patients with autoimmune diseases, malignant tumors, severe infections, or severe liver or kidney dysfunction, as these conditions independently affect metabolism and would confound the NRP-specific metabolic signature, and (4) patients without complete clinical examination and imaging data. The flow chart of this study was summarized in Figure 1.

### Neuroimaging examination and analysis

All enrolled patients with AIS underwent neuroimaging examinations, including baseline cerebral CTP, carotid-to-vertex CT angiography, and 24-h follow-up cerebral CTP or MRI dynamic susceptibility contrast-enhanced perfusion imaging (MRP). CTP and MRP were processed using the RAPID software (iSchemaView, Menlo Park, CA) to generate relative cerebral blood volume (rCBV), relative cerebral blood flow (rCBF), and mean transit time (MTT) maps. Baseline CTP was co-registered with follow-up diffusion-weighted imaging (DWI) and MRP or unenhanced CT and CTP. Mirror homologous contralateral hemispheres were created to adjust for anatomical variations in gray/white matter composition among participants.

The no-reflow phenomenon (NRP) was defined according to previously published criteria (9). On 24-h post-EVT CTP, NRP was characterized by persistent hypoperfusion within the infarct territory, manifest as reduced rCBV or rCBF. Two neurologists, blinded to all clinical data, performed qualitative assessment; quantitative confirmation required an asymmetry >15% between the median rCBV or rCBF value of the target lesion and the mean value of the corresponding contra-lateral mirror region. This 15% threshold accounts for the physiological inter-hemispheric perfusion difference observed in healthy subjects (~14.5%) and the asymmetry measured in non-ischaemic brain areas of stroke patients undergoing MRP (~15%), thereby excluding normal physiological variation. Disagreements between the two reviewers were resolved by consensus discussion.

### Clinical examination

Clinical data for all participants were collected from medical records. The clinical laboratory tested white blood cells, neutrophils, monocytes, lymphocytes, red blood cells, platelets, glucose, fibrinogen, prothrombin time, D-dimer, lifestyle factors (smoking, alcohol consumption), and vascular risk factors (hypertension, type 2 diabetes, hyperlipidemia, coronary heart disease, cerebral hemorrhage, atrial fibrillation, atherosclerosis). The severity of stroke was assessed using the National Institutes of Health Stroke Scale (NIHSS) upon admission. Short-term outcomes were measured based on NIHSS scores after emergency stroke surgery.

### Sample preparation

Peripheral blood was collected in EDTA-containing tubes within 24 h after emergency endovascular thrombectomy. Samples were centrifuged at 3,000 rpm for 15 min at 4 °C, and the resulting plasma was stored at −80 °C until analysis. The collected samples were thawed on ice, and metabolites were extracted with 80% methanol buffer. Briefly, 100 μL of sample was extracted with 400 μL of precooled methanol. The extraction mixture was then stored at −20 °C for 30 min. After centrifugation at 20,000 g for 15 min, the supernatants were transferred into new tubes to and vacuum-dried. The samples were redissolved with 100 μL 80% methanol and stored at −80 °C prior to LC–MS analysis. In addition, pooled QC samples were also prepared by combining 10 μL of each extraction mixture.

### LC-HRMS analysis

All samples were analyzed using an LC-HRMS system according to the instrument instructions. Chromatographic separation was performed using an UltiMate 3000 UPLC system (Thermo Fisher Scientific, Bremen, Germany). An ACQUITY UPLC T3 column (100 mm × 2.1 mm, 1.8 μm, Waters, Milford, United States) was used for reverse-phase separation. The column was maintained at 40 °C with a mobile phase consisting of solvent A (5 mM ammonium acetate and 5 mM acetic acid) and solvent B (acetonitrile). The flow rate was set at 0.3 mL/min. The gradient elution conditions were as follows: 0–0.8 min, 2% B; 0.8–2.8 min, 2–70% B; 2.8–5.6 min, 70–90% B; 5.6–6.4 min, 90–100% B; 6.4–8.0 min, 100% B; 8.0–8.1 min, 100–2% B; 8.1–10 min, 2% B.

Q-Exactive high-resolution tandem mass spectrometer (Thermo Scientific) was used to detect metabolites eluted from the column. The Q-Exactive operated in both positive and negative ion modes. Metabolite precursor spectra were collected at a resolution of 70,000 (m/z 70–1,050) with an AGC target of 3e6. The maximum injection time was set to 100 ms. Data acquisition was performed in DDA mode with a top 3 configuration. Fragment spectra were collected at a resolution of 17,500 with an AGC target of 1e5 and a maximum injection time of 80 ms. To assess the stability of LC–MS throughout the acquisition process, a quality control sample (mixed sample) was collected after every 10 samples.

Q-Detailed parameters for metabolite extraction, liquid chromatography, and mass spectrometry are provided in Supplementary Methods S1.

### Data processing and statistical analysis

Untargeted metabolomic data processing was performed using XCMS software to preprocess the mass spectrometry data (17, 18), including peak picking, peak grouping, retention time correction, secondary peak grouping, isotope and adduct labeling. The raw LC–MS data files were converted into mzXML format and then processed using the XCMS, CAMERA (19), and metaX (20) toolboxes implemented in R software. Ions were identified based on retention time (RT) and m/z data. The intensity of each peak was recorded, and a three-dimensional matrix was generated containing the peak index (RT-m/z pair), sample name (observation value), and ion intensity information (variable). Metabolites were annotated by matching the precise molecular mass data (m/z) of the samples with those in the online KEGG and HMDB databases. If the mass difference between the observed value and the database value was less than 10 ppm, the metabolite was annotated and further identified and validated by isotope distribution measurement. We also used an in-house database to verify metabolite identification. Statistical analysis of clinical characteristics was performed with SPSS 26.0 (IBM, Armonk, NY, United States). Continuous variables with normal distribution are expressed as mean ± SD and compared using Student’s t-test. Spearman’s rank correlation was employed for association analyses. The metabolomics data matrix was log-transformed and auto-scaled (mean-centering + UV scaling). Multivariate modeling: group differences were examined by orthogonal partial least-squares discriminant analysis, permutation test with 100 permutations (OPLS-DA). ROC curves were constructed using the pROC package (version 1.18.0) in R software (version 4.2.1). For each metabolite, sensitivity and specificity were calculated across all possible threshold values, and the AUC was computed using the trapezoidal rule with 95% confidence intervals estimated by 2,000 bootstrap resamples (21, 22). Optimal cut-off points were determined using Youden’s index (sensitivity + specificity − 1). DeLong’s test was used to compare AUCs between different metabolites. Metabolites with AUC > 0.7 and lower bound of 95% CI > 0.5 were considered potentially useful discriminators.

## Results

### Clinical characteristics and imaging findings of the study population

The baseline characteristics of the patients are presented in Table 1. Pre-procedure NIHSS scores were comparable between groups (NRP 12.9 ± 3.1 vs. RP 12.8 ± 2.9, *p* = 0.92). Post-procedure NIHSS differed significantly (NRP 11 (9–13) vs. RP 7 (5–9), *p* = 0.025); higher scores predicted worse outcomes. Haemorrhagic transformation occurred in 60% of NRP patients versus 1% of RP patients (*p* = 0.001). Laboratory analyses revealed higher leukocyte counts in the NRP group (*p* = 0.015) and a marked elevation in neutrophils (*p* = 0.006).

**Table 1:** Baseline characteristics of all patients.

Variables	Entire population (*n* = 34)		*p* value
	RP group (*n* = 19)	NRP group (*n* = 15)	
Demographics			
Male, *n* (%)	11.0 (57.9)	12.0 (80.0)	0.350
Age (years), mean (±SD)	64.4 (14.8)	63.3 (13.1)	0.784
Vascular risk factors			
Hypertension, *n* (%)	9.0 (47.4)	6.0 (40.0)	0.667
Diabetes mellitus, *n* (%)	5.0 (26.3)	4.0 (26.7)	0.982
Coronary artery disease, *n* (%)	3.0 (15.8)	0 (0)	0.107
Arteriosclerosis, *n* (%)	7.0 (36.8)	1.0 (6.7)	0.039*
Atrial fibrillation, *n* (%)	8.0 (42.1)	4.0 (26.7)	0.350
Hyperlipoidemia, *n* (%)	7.0 (36.8)	4.0 (26.7)	0.529
Smoking, *n* (%)	10.0 (52.6)	10.0 (66.7)	0.409
Drinking, *n* (%)	7.0 (36.8)	6.0 (40.0)	0.851
Clinical characteristics			
Admission NIHSS, mean (±SD)	12.8 (4.8)	12.9 (5.1)	0.973
Post NIHSS, median (IQR)	7.0 (5.0–8.0)	11.0 (9.0–13.0)	0.025*
Laboratory tests			
WBCs (×10^9^/L), mean (±SD)	7.3 (2.1)	10.3 (4.4)	0.015*
Neutrophils (×10^9^/L), median (IQR)	4.8 (3.4–5.9)	7.1 (5.5–10.6)	0.006**
Monocytes(×10^9^/L), median (IQR)	0.6 (0.4–0.8)	0.6 (0.5–1.0)	0.157
Lymphocytes(×10^9^/L), mean (±SD)	1.4 (0.5)	1.4 (0.8)	0.864
RBCs(×10^9^/L), mean (±SD)	4.2 (0.8)	4.0 (0.7)	0.515
PLT (×10^9^/L), median (IQR)	216.0 (191.0–285.0)	257.0 (210.0–364.0)	0.083
Glucose (mmol/L), median (IQR)	5.8 (5.2–7.2)	6.2 (5.5–8.2)	0.319
Fib (g/L), median (IQR)	3.6 (2.6–4.8)	4.4 (3.8–5.7)	0.089
INR, median (IQR)	1.0 (1.0–1.1)	1.0 (1.0–1.1)	0.256
D-dimer (mg/L), median (IQR)	1.1 (0.6–2.7)	2.0 (0.7–8.5)	0.147
Hemorrhage transformation, *n* (%)	1 (5.3%)	9 (60.0%)	0.001***

RP, Reflow phenomenon; NRP, no-reflow phenomenon; NIHSS, National Institutes of Health Stroke Scale; WBCs, white blood cells; PLT, platelets; Fib, fibrinogen (*p < 0.05, **p < 0.01, ***p < 0.001).

Baseline medication history was comparable between the two groups. Detailed information on medication use, including statistical comparisons between RP and NRP patients, is provided in Supplementary Table S1. No statistically significant differences were observed between the two groups in any medication category (all *p* > 0.05).

### Representative CTP cases of no-reflow

**Figure 2:**
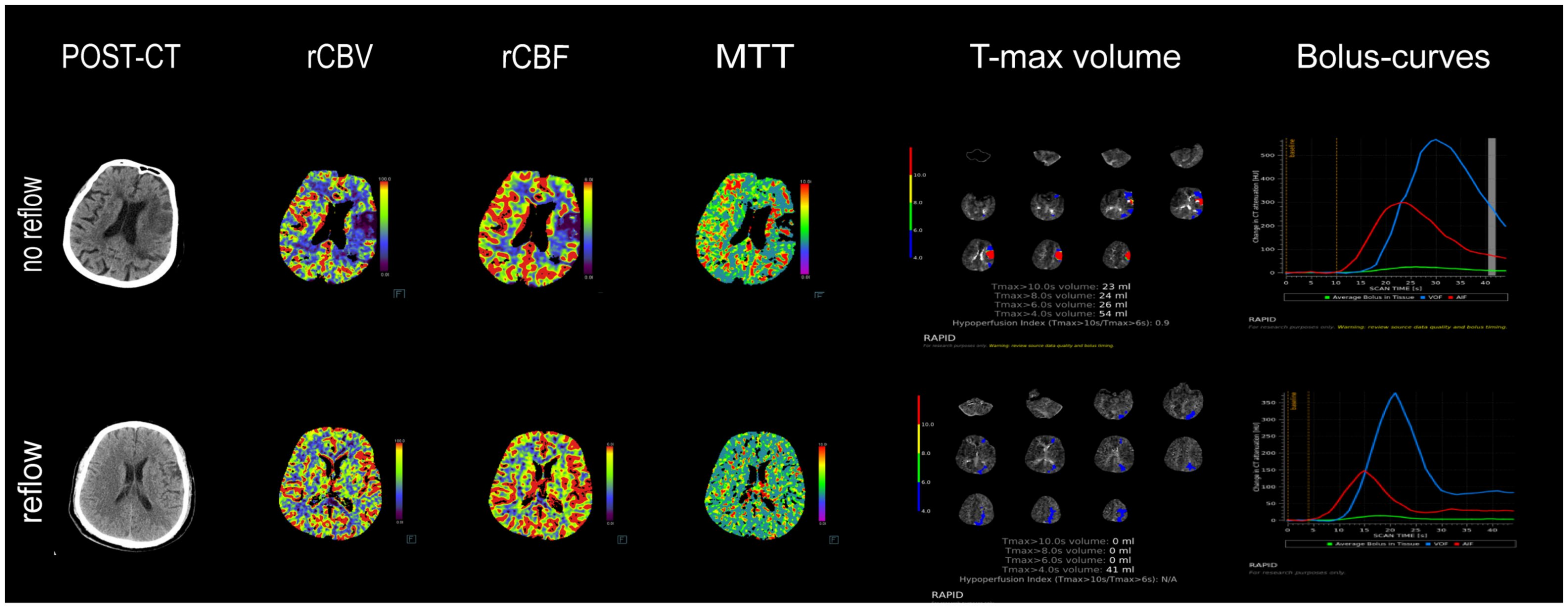
Representative CT perfusion cases of with and without reflow. No-reflow identified on CT perfusion and normal follow-up perfusion imaging for comparison.

Infarct location was first localized on contrast-enhanced CT, after which perfusion asymmetry within the corresponding territory was evaluated on the parametric maps. In no-reflow (NRP) cases, relative cerebral blood volume (rCBV) and relative cerebral blood flow (rCBF) were reduced within the infarct, while mean transit time (MTT) was markedly prolonged. Nevertheless, the arterial input function (AIF) and venous output function (VOF) contrast bolus curves remained normal (x-axis = scan time, y-axis = Hounsfield units; red line = AIF, blue line = VOF, green line = mean tissue curve). The normal AIF and VOF contrast bolus curves in both groups indicate that large-vessel inflow and venous outflow were not compromised (23, 24). This confirms that the observed perfusion deficits in NRP patients were attributable to microvascular dysfunction rather than proximal arterial input problems or venous outflow obstruction, consistent with the pathophysiological definition of no-reflow (Figure 2).

Due to technical factors during image acquisition and post-processing, we focused on two well-established perfusion parameters including Tmax > 6 s and rCBF < 30%, which are widely used in acute ischemic stroke for hypoperfusion assessment (25). Quantitative analysis was performed using RAPID software, and detailed perfusion data are provided in Supplementary Figure S1.

### Multivariate statistical analysis of plasma metabolomics in patients with and without reflow

**Figure 3:**
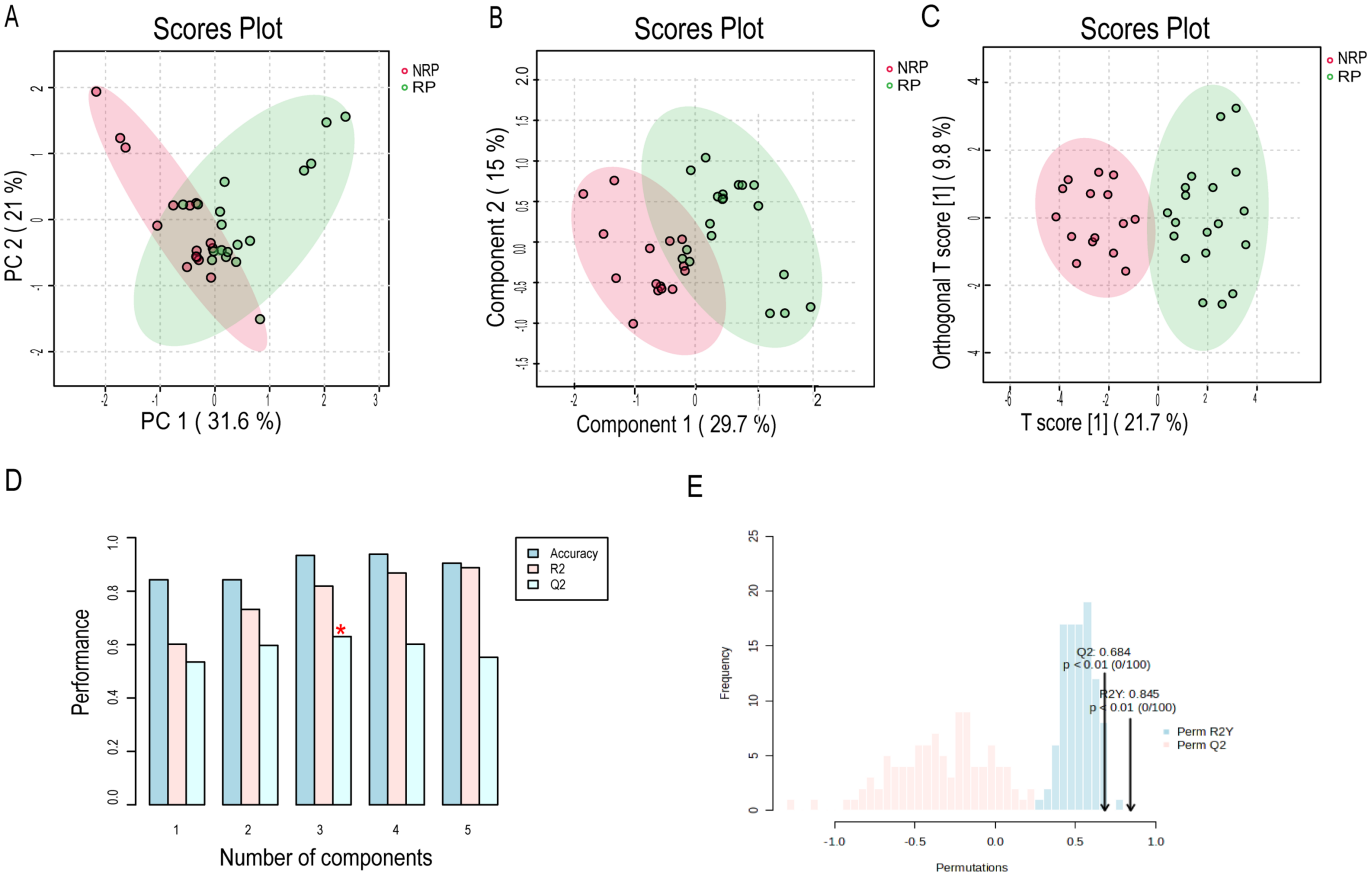
Multivariable statistical analysis and pathway analysis between AIS patients with RP and NRP. **(A)** Principal component analysis (PCA) score plots; **(B)** projections to latent structures-discriminant analysis (PLS-DA) score plots; **(C)** orthogonal projections to latent structures—discriminant analysis (OPLS-DA) score plots; **(D)** performance metrics (accuracy, R^2^, Q^2^) for OPLS-DA models with increasing numbers of components. The red asterisk indicates the position of the original model’s *Q*^2^ value within the permutation test distribution, confirming that the model is not overfitted (*R*^2^*Y* = 0.845, *Q*^2^ = 0.684, accuracy = 0.9333); **(E)** permutation test of OPLS-DA (*R*^2^*Y* = 0.845, *Q*^2^ = 0.684).

**Figure 4:**
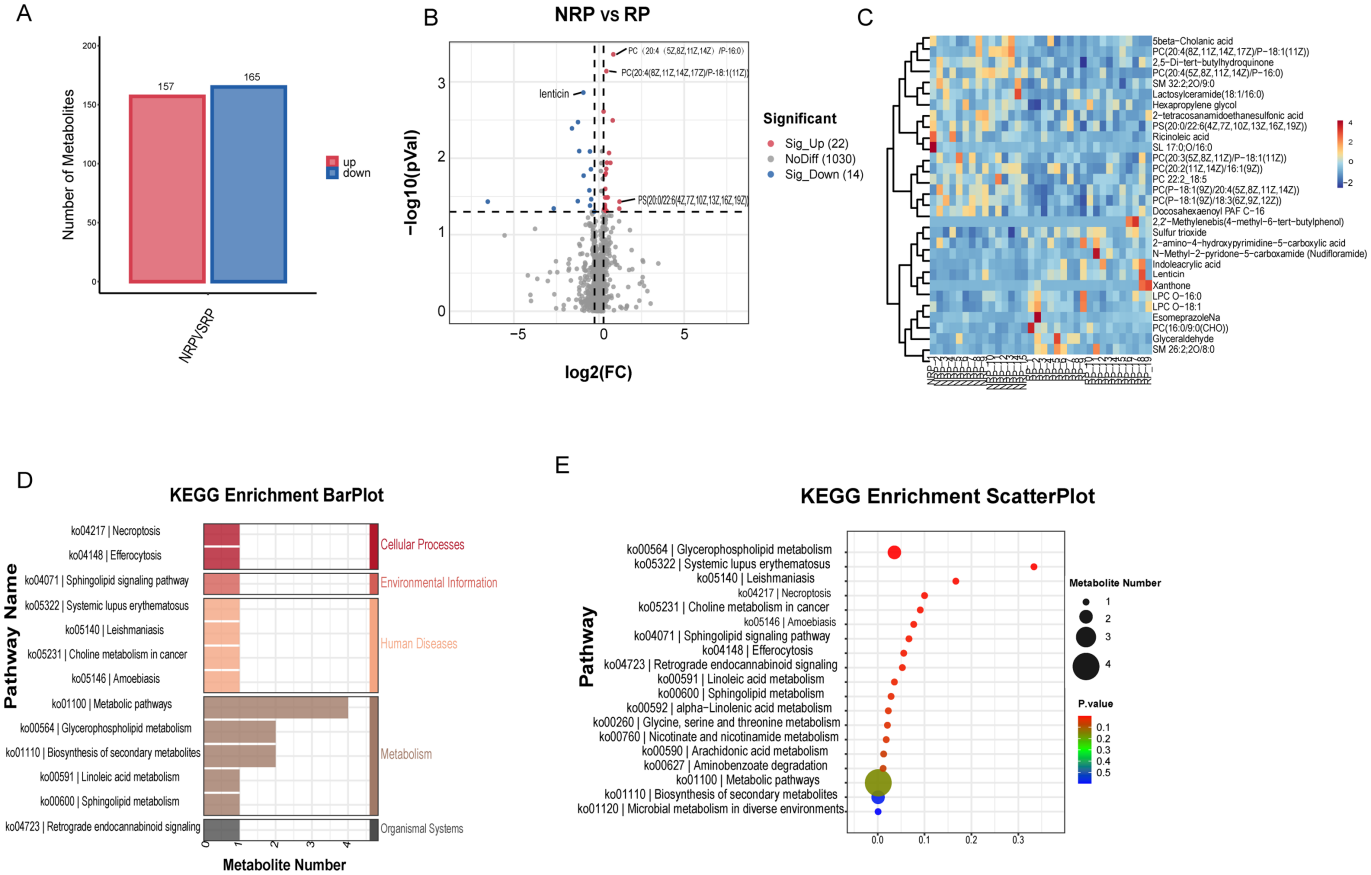
Identification of the differential metabolomic profiles between the RP groups and NRP groups. **(A)** A total of 322 metabolites. **(B)** Volcano plot was created for all metabolites in the differential expression analysis. **(C)** The top 30 differentially expressed metabolites are displayed in a heatmap. **(D)** KEGG enrichment barplot; **(E)** KEGG enrichment pathway.

Multivariate statistical analysis of plasma metabolomics (Figures 3B,C) and differential metabolite identification (Figures 4A, C,D) were performed between patients with and without reflow.

#### Principal component analysis

Principal component analysis (PCA) was used for unsupervised pattern recognition of the preprocessed metabolites. The PCA score plot in Figure 3A showed a clear separation between the NRP and RP groups along the PC1 direction, indicating significant differences between the two groups in the first principal component. Although the differences in the PC2 direction were smaller, the two groups were distinctly separated in the principal component space overall.

#### Partial least squares discriminant analysis

Supervised multivariate statistical analysis was performed using partial least squares discriminant analysis (PLS-DA) to analyze the differences between the two groups. The model achieved *R*^2^*Y* = 0.753 and *Q*^2^ = 0.698. The score plot and cross-validation results are shown in Figure 3D. Values of *R*^2^ and *Q*^2^ closer to 1 indicate a better PLS-DA model.

#### Orthogonal partial least squares discriminant analysis

An orthogonal partial least squares discriminant analysis (OPLS-DA) model was constructed with *R*^2^*Y* = 0.845 and *Q*^2^ = 0.684. The score plot is shown in Figure 3E. The model effectively distinguished between reflow and no-reflow, with strong explanatory and predictive power. The OPLS-DA model achieved *R*^2^*Y* = 0.845, indicating that 84.5% of the variance in group membership (RP vs. NRP) can be explained by the metabolite profiles (strong explanatory power). The *Q*^2^ = 0.684 represents the model’s predictive ability estimated through 7-fold cross-validation; values > 0.5 are considered good, and >0.9 excellent. Thus, our *Q*^2^ of 0.684 indicates that the model has moderately strong predictive power for distinguishing NRP from RP based on metabolomic profiles.

### Plasma metabolomic signature distinguishing reperfused from no-reflow AIS patients

A total of 322 plasma metabolites were confidently identified and quantified across RP and NRP patients. Among them, 29 metabolites reached statistical significance (VIP > 1, *p* < 0.05) between the AIS subgroups (Supplementary Table S2). Of these discriminatory metabolites, 12 were down-regulated (fold change < 1) and 17 were up-regulated (fold change > 1). A volcano plot was created to visualize the overall distribution of differential metabolites. It is important to note that the heatmap uses Z-score normalization, allowing for horizontal comparison of the same metabolite across different samples but not vertical comparison of different metabolites (Figure 4B).

The KEGG IDs of the differential metabolites were input into MetaboAnalyst 6.0 for metabolic pathway analysis, and a pathway bubble plot was generated (Figure 4E). Our results showed significant differences in glycerophospholipid metabolism, linoleic acid metabolism, α-linolenic acid metabolism, and niacin and nicotinamide metabolism pathways between patients with and without reflow. Among these, glycerophospholipid metabolism had the strongest discriminative ability between the two groups (*p* < 0.01) and was the most significantly altered metabolic pathway (impact value of 0.140) (Table 2).

**Table 2:** KEGG differential pathway analysis between AIS patients with and without reflow.

Pathway name	Match status	*p* value	−log(p)	FDR	Impact
Glycerophospholipid metabolism	2/36	0.0029003	2.5376	0.23203	0.13988
Linoleic acid metabolism	1/3	0.0125150	1.9026	0.50062	0
Alpha-linolenic acid metabolism	1/15	0.0322950	1.4909	0.74387	0

### Plasma metabolites with potential diagnostic value for no-reflow

**Figure 5:**
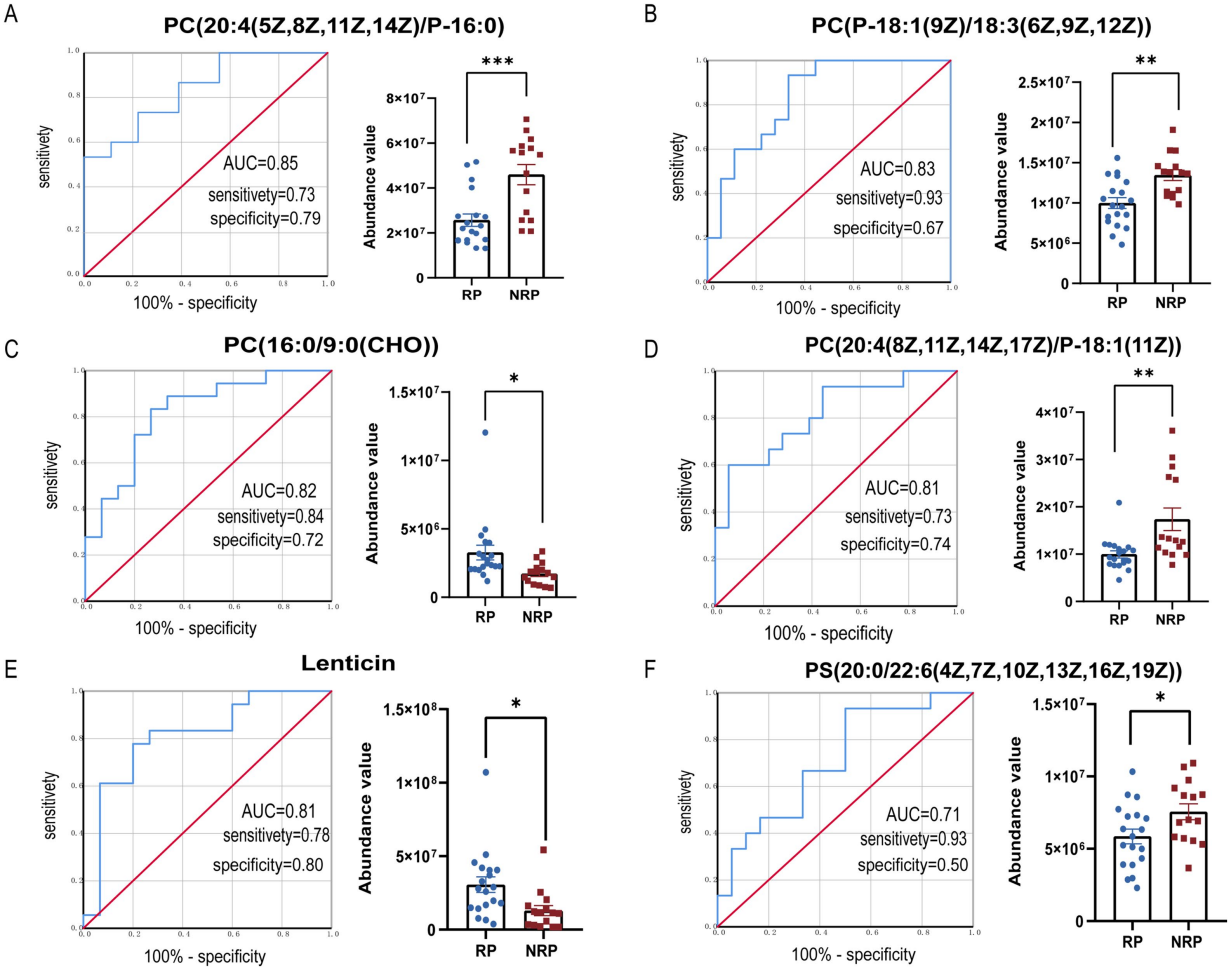
Identification of potential metabolite biomarkers of NRP. **(A)** Comparison and ROC analysis for PC(20:4(5Z,8Z,11Z,14Z)/P-16:0). **(B)** PC(P-18:1(9Z)/18:3(6Z,9Z,12Z)). **(C)** PC(16:0/9:0(CHO)). **(D)** PC(20:4(8Z,11Z,14Z,17Z)/P-18:1(11Z)). **(E)** Lenticin. **(F)** PS(20:0/22:6(4Z,7Z,10Z,13Z,16Z,19Z)) (*p < 0.05, **p < 0.01, ***p < 0.001).

To identify metabolites that could serve as biomarkers of NRP, ROC curves were constructed for all 29 significantly differential metabolites, and the area under the ROC curve (AUC), specificity, and sensitivity were calculated to evaluate their ability to distinguish between patients with successful reperfusion and those with no-reflow. The results showed that five metabolites had an AUC > 0.8, most of which were members of the PC family (KEGG ID: C00157). Phosphatidylserine (KEGG ID: C02737) belongs to the glycerophospholipid metabolism pathway and is also involved in the biosynthesis of phosphatidylcholine and phosphatidylethanolamine. ROC curve analysis was used to evaluate the potential of differential metabolites as discriminative biomarkers for distinguishing NRP from RP in AIS patients who have undergone successful thrombectomy (Figure 5). The relative abundances of the differential metabolites are provided in Supplementary Figure S2. Complete diagnostic efficacy indicators, including AUC, sensitivity, specificity, and fold change for each metabolite, are provided in Supplementary Table S2.

### Correlation analysis of differential metabolites with clinical indicators

**Figure 6:**
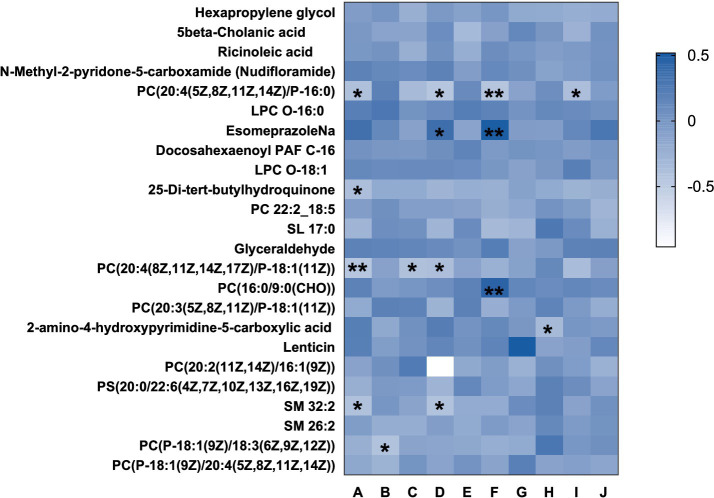
This figure is a heatmap illustrating the correlation strength between multiple metabolites and ten hematological parameters. Each cell’s color or numeric value represents the Pearson correlation coefficient, ranging from −0.5 (strong negative correlation) to +0.5 (strong positive correlation), with values near zero indicating no correlation. A, WBC (10^9^/L), B, RBC (10^12^/L), C, PLT (10^9^/L), D, NEU (10^9^/L), E, L (10^9^/L) F, M (10^9^/L), G, Glu (mmol/L), H, LDL (mmol/L), I, INR(s), J, D-Dimer (mg/L) (*p < 0.05, **p < 0.01).

We performed correlation analysis between the 29 differential metabolites and clinical indicators in patients with and without reflow. Among the 29 differentially expressed metabolites, 24 were included in the correlation analysis with clinical parameters; the remaining 5 metabolites were excluded due to low detection rate in certain samples. The complete list of these metabolites, including their HMDB and KEGG IDs, retention times, and VIP scores, is provided in Supplementary Table S3. Since metabolite abundance and clinical measurements were not normally distributed, Spearman rank correlation analysis was used, including White Blood Cell, White Blood Cell, Platelet, Red Blood Cell, Lymphocyte, Monocyte, Glucose, Low-DensityLipoprotein, Fibrinogen, International Normalized Ratio. The results are shown in Figure 6. Spearman rank correlation analysis was used to assess associations between the 24 differential metabolites and clinical indicators (WBC, NEU, PLT, RBC, LYM, MON, Glu, LDL, INR, D-dimer). Key findings include: PC(20:4(5Z,8Z,11Z,14Z)/P-16:0) was negatively correlated with RBC (*r* = −0.340, *p* = 0.049), MON (*r* = −0.439, *p* = 0.009), and INR (*r* = −0.377, *p* = 0.028), and positively correlated with NEU (*r* = 0.364, *p* = 0.034). PC(22:2_18:5) was negatively correlated with glucose (*r* = −0.547, *p* = 0.001). 2,5-Di-tert-butylhydroquinone exhibited inverse correlations with WBC (*r* = −0.359, *p* = 0.037) and NEU (*r* = −0.414, *p* = 0.015). PC(16:0/9:0(CHO)) was inversely associated with NEU (*r* = −0.381, *p* = 0.026) and positively with MON (*r* = 0.451, *p* = 0.007). PC(20:4(8Z,11Z,14Z,17Z)/P-18:1(11Z)) was negatively correlated with RBC (*r* = −0.401, *p* = 0.019) and INR (*r* = −0.354, *p* = 0.040). Lenticin was inversely associated with NEU (*r* = −0.384, *p* = 0.025). PS(20:0/22:6) was positively correlated with NEU (*r* = 0.396, *p* = 0.021). Among all clinical indicators, neutrophils count (NEU) demonstrated the strongest and most consistent correlations with differential metabolites. The complete correlation matrix, including correlation coefficients and *p*-values for all metabolite-clinical parameter pairs, is provided in Supplementary Table S4.

## Discussion

This study employed non-targeted liquid chromatography-high resolution mass spectrometry (LC-HRMS) metabolomics to identify plasma metabolic signatures that distinguish acute ischemic stroke (AIS) patients who develop no-reflow phenomenon (NRP) despite successful endovascular recanalization. Currently, NRP diagnosis primarily relies on advanced neuroimaging, which is costly, time-consuming, and limited by geographical resources. In contrast, plasma metabolomics offers a minimally invasive detection method capable of capturing systemic metabolic changes during the acute phase of stroke, providing insights into the underlying pathophysiological processes and complementing neuroimaging findings. The study detected 29 significantly altered metabolites, predominantly phosphatidylcholines (PCs). Glycerophospholipid metabolism emerged as the significantly perturbed pathway, suggesting its disruption may be involved in NRP pathogenesis (26).

Plasma levels of PC (20:4(5Z,8Z,11Z,14Z)/P-16:0), PC (P-18:1(9Z)/18:3(6Z,9Z,12Z)), and PC (20:4(8Z,11Z,14Z,17Z)/P-18:1(11Z)) were significantly elevated in the NRP group, whereas PC (16:0/9:0(CHO)) was markedly decreased (27). The prominently elevated PCs often contain arachidonic acid. Such lipids may originate from cell membrane damage following activation of phospholipase A₂, reflecting early activation of inflammatory responses and potentially participating in vasoconstriction and leukocyte adhesion, thereby affecting microcirculatory perfusion (28, 29). The decreased level of PC(16:0/9:0(CHO)), an oxidatively modified lipid, might suggest early oxidative products are gradually cleared within 24 h (30). However, this clearance process might be insufficient in NRP patients. Reactive oxygen species-mediated microvascular dysfunction is a key contributor to no-reflow, and the observed alterations in oxidized phospholipids may reflect this underlying pathological process (31).

In this study, we also found that plasma phosphatidylserine (PS) levels were significantly elevated in the NRP group. Of note, studies have suggested that exogenous PS supplementation exerts neuroprotective effects (32). This discrepancy may arise from the distinct roles of PS at different stages (33). In the acute phase of stroke, PS exposed on activated platelets and stressed cells mainly exerts pro-inflammatory and pro-coagulant effects, exacerbating microvascular no-reflow (34, 35). In contrast, during the subacute or chronic phase, PS exposed on the surface of apoptotic neurons mediates efferocytosis to clear cellular debris and provides signals for axonal regeneration, thereby creating a favorable environment for neural repair (33).

Conversely, levels of Lenticin were higher in the reperfusion (RP) group. Research suggests this substance possesses anti-inflammatory and insulin-sensitizing properties (36, 37), its elevation in the RP group might reflect a protective response, which appears lacking in the NRP group.

The prominent alterations in glycerophospholipid metabolism in NRP underscore its dual function in maintaining membrane integrity and regulating inflammatory signaling. Disruption of PC and PS metabolism could compromise blood–brain barrier stability and amplify pro-thrombotic activity (38). Our pathway analysis (impact value = 0.140, *p* < 0.01), combined with existing animal studies showing that glycerophospholipid supplementation can reduce infarct size following ischemia–reperfusion injury, suggests this pathway warrants further in-depth investigation (39, 40).

Beyond these pathway-level findings, we also preliminarily explored correlations between differential metabolites and clinical parameters, which may offer clues to underlying pathophysiological processes.

Results showed consistent associations between neutrophils (NEU) and several metabolites. NEU positively correlated with PC(20:4/P-16:0) (*r* = 0.364, *p* = 0.034), which can be released by activated neutrophils, consistent with reports of neutrophil involvement in microvascular obstruction and inflammation in NRP (41). NEU also positively correlated with PS (20:0/22:6) (*r* = 0.396, *p* = 0.021), a marker of apoptosis and procoagulant activity, suggesting neutrophil-related oxidative stress might influence coagulation status.

Furthermore, PC (20:4/P-16:0) and PC (20:4/P-18:1) were negatively correlated with red blood cell (RBC) count (*r* = −0.340 and −0.401, respectively). In NRP patients, microcirculatory disturbances may expose RBCs to oxidative damage, leading to shedding of membrane phospholipids. A strong negative correlation was found between glucose and PC (22:2_18:5) (*r* = −0.547, *p* = 0.001), suggesting hyperglycemia might accelerate membrane phospholipid degradation (16). International normalized ratio (INR) also negatively correlated with two ether-linked phospholipids (*r* = −0.377 and −0.354), implying depletion of these anticoagulant phospholipids might favor microvascular thrombosis. Collectively, these exploratory associations suggest interactions among neutrophil activation, oxidative stress, and coagulation status in NRP. Compared to single clinical parameters, metabolites might integrate these processes more comprehensively, though causality requires experimental validation.

The relatively high incidence of no-reflow phenomenon in our cohort (44%) may relate to the inclusion of patients with anterior circulation large vessel occlusion, early (<24 h) perfusion assessment, and stringent NRP diagnostic criteria (42). The higher rate of hemorrhagic transformation in the NRP group is noteworthy and might be partly associated with their higher post-procedural NIHSS scores. Blood–brain barrier disruption leading to cerebral edema could exacerbate no-reflow caused by vascular obstruction (43), and conversely, no-reflow might further aggravate endothelial injury, disrupting the blood–brain barrier (44). However, the specific mechanisms require further investigation. Additionally, the significant elevation of neutrophils in no-reflow patients aligns with previous reports, confirming the important role of neutrophils in NRP development.

This study has several limitations. First, the sample size is relatively small and from a single center, limiting the generalizability of the findings. Second, we acknowledge that patients with significant ipsilateral carotid stenosis or contralateral anterior circulation disease were not excluded from this study. Third, blood samples were collected at only one time point (24 h post-procedure), which fails to capture dynamic metabolic changes and precludes causal inference. Furthermore, although efforts were made to control for confounders, potential interfering variables such as medication history could not be fully incorporated. Finally, the identified differential metabolites require functional validation through basic experiments and confirmation of reliability using targeted metabolomics approaches in multicenter cohorts.

## Conclusion

This study utilized untargeted LC–MS metabolomics to profile plasma metabolites in acute ischemic stroke patients who underwent thrombectomy with successful vascular recanalization. Our findings show that certain plasma metabolites, particularly phosphatidylcholines, differ between patients with and without the no-reflow phenomenon. These differences point to oxidative stress-related alterations in membrane phospholipids, with the glycerophospholipid metabolism pathway emerging as a possible focus for future mechanistic studies. The identified biomarkers demonstrate promising discriminative ability and may contribute to early identification of no-reflow, offering a potential avenue for developing strategies to improve reperfusion therapy in stroke.

## Data Availability

The original contributions presented in the study are included in the article/Supplementary material, further inquiries can be directed to the corresponding authors.
