# Supplementary material for: Plasma metabolomic signatures of the no-reflow phenomenon in stroke patients following thrombectomy
Source: Front Neurol. 2026 Jun 19;17:1740882. doi: 10.3389/fneur.2026.1740882 (PMC13330104; doi:10.3389/fneur.2026.1740882)
Supplement: Supplementary file 1 [file Supplementary_file_1.docx]

Supplementary Material

# Supplementary Figure1. Comparison of Cerebral Perfusion Parameters Between Patients with Successful Reperfusion (RP) and No-Reflow (NRP).

#
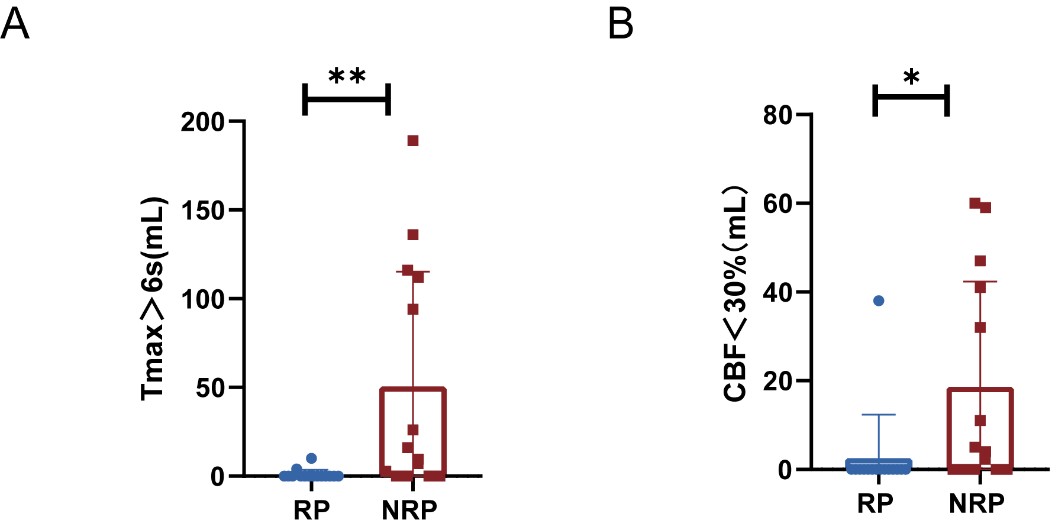


**(A)** Quantitative analysis of the volume of regions with Tmax > 6 seconds (mL) in the RP (reperfusion) and NRP (no-reflow) groups. Data are presented as box plots with individual data points. The NRP group exhibited a significantly larger volume of Tmax > 6 seconds compared with the RP group (******, P < 0.01).

**(B)** Quantitative analysis of the volume of regions with cerebral blood flow (CBF) < 30% (mL) in the RP and NRP groups. The NRP group demonstrated a significantly expanded area of severe hypoperfusion (CBF < 30%) relative to the RP group (*, P < 0.05).

Abbreviations: RP, reperfusion; NRP, no-reflow; Tmax, time to maximum; CBF, cerebral blood flow.

# Supplementary Figure2.Differential metabolites are shown in box plots. The purple group represents the NRP group, and the red group represents the RP group.

#
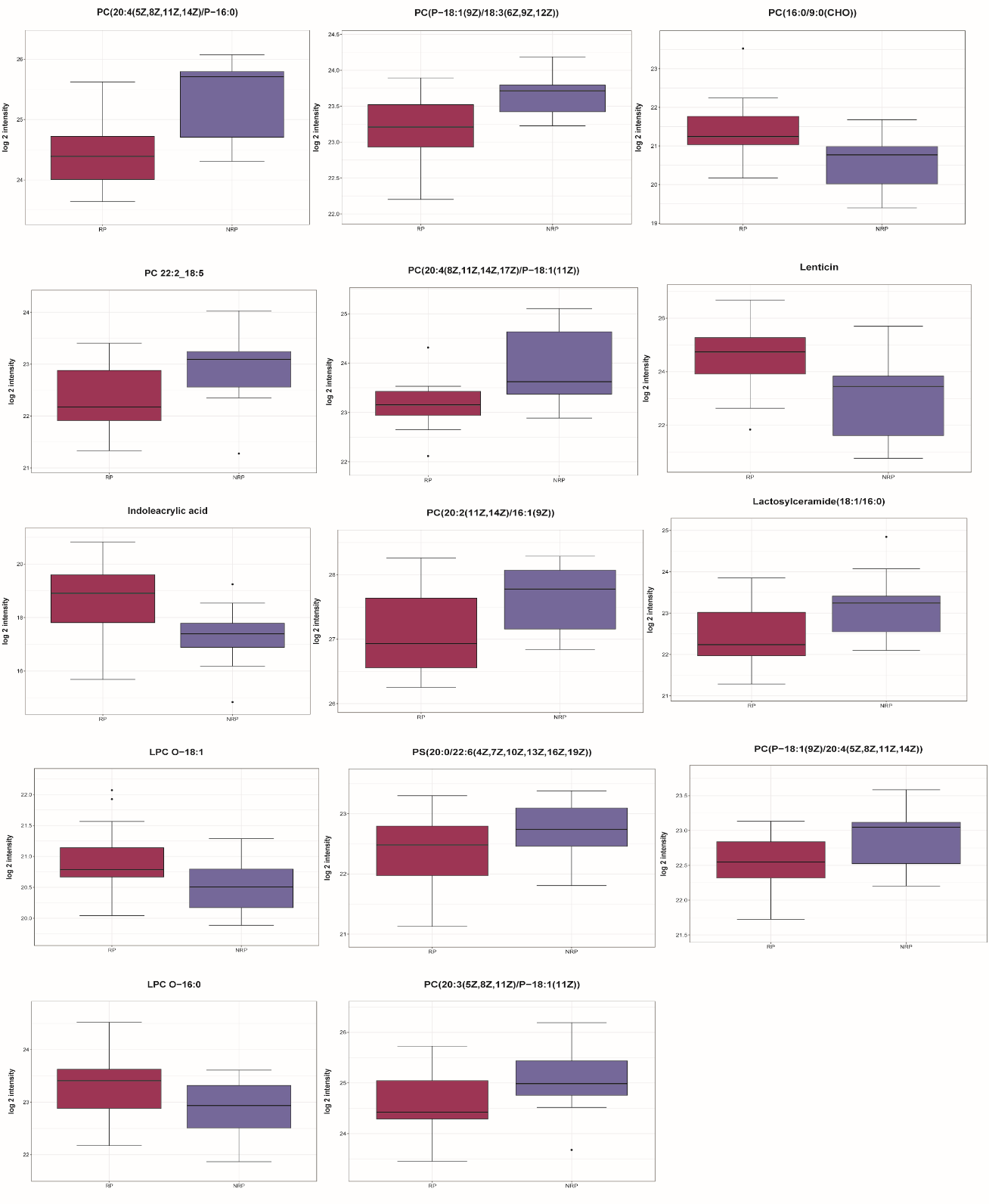


**Supplementary Table 1.** Baseline Medication History of Patients in the RP and NRP Groups

| **Medication History， n (%)** | **RP group(n=15)** | **NRP group (n=15)** | **P value** |
| --- | --- | --- | --- |
| **Statins** | 2 (13.3) | 1 (6.7) | 0.543 |
| **Lipid-Lowering Agents** | 2 (16.7) | 1(6.7) | 0.411 |
| **Antihypertensive Agents** | 7 (58.3) | 6 (40.0) | 0.343 |
| **Antidiabetic Agents** | 4 (33.3) | 1(6.7) | 0.076 |
| **Anticoagulant Agents** | 2 (16.7) | 1(6.7) | 0.411 |
| **Thrombolysis Agents** | 3(25.0) | 3(20.0) | 0.756 |

Data are presented as number (percentage). P values were calculated using the chi-square test.
RP, reflow phenomenon; NRP, no-reflow phenomenon.

# Supplementary Table2. Blood biomarkers for the diagnosis of no reflow phenomenon (NRP).

| **Metabolite** | **AUC** | **95% CI**  **of AUC** | **Sensitivity** | **Specificity** | **Fold change** | **VIP** | **Up/Down** |
| --- | --- | --- | --- | --- | --- | --- | --- |
| PC(20:4(5Z,8Z,11Z,14Z)/P-16:0) | 0.85 | 0.717-0.974 | 0.733 | 0.789 | 1.79 | 2.64 | up |
| PC(P-18:1(9Z)/18:3(6Z,9Z,12Z)) | 0.83 | 0.692-0.964 | 0.933 | 0.667 | 1.35 | 1.8 | up |
| PC(16:0/9:0(CHO)) | 0.82 | 0.671-0.964 | 0.84 | 0.722 | 0.53 | 2.43 | down |
| PC(20:4(8Z,11Z,14Z,17Z)/P-18:1(11Z)) | 0.81 | 0.663-0.958 | 0.73 | 0.737 | 1.75 | 2.6 | up |
| PC(20:2(11Z,14Z)/16:1(9Z)) | 0.75 | 0.588-0.914 | 0.73 | 0.632 | 1.38 | 1.73 | up |
| PC 22:2_18:5 | 0.74 | 0.568-0.92 | 0.87 | 0.632 | 1.50 | 1.85 | up |
| PC(18:3(9Z,12Z,15Z)/P-18:1(11Z)) | 0.73 | 0.553-0.907 | 0.73 | 0.684 | 1.29 | 1.24 | up |
| PC(P-18:1(9Z)/20:4(5Z,8Z,11Z,14Z)) | 0.72 | 0.534-0.898 | 0.67 | 0.632 | 1.29 | 1.40 | up |
| PC(20:3(5Z,8Z,11Z)/P-18:1(11Z)) | 0.71 | 0.535-0.89 | 0.87 | 0.579 | 1.38 | 1.45 | up |
| 3E-tetradecenoic acid | 0.71 | 0.521-0.889 | 0.67 | 0.63 | 1.36 | 1.36 | down |
| PS(20:0/22:6(4Z,7Z,10Z,13Z,16Z,19Z)) | 0.70 | 0.524-0.879 | 0.93 | 0.474 | 1.29 | 1.4 | up |

AUC: Area under the ROC curve; CI: Confidence interval; OR: Odds ratio.

**Supplementary Table 3.** Differential metabolites in patients with and without reflow via untargeted metabolomic.

| **Metabolites** | **MZ** | **RT** | **HMDB** | **KEGG** | **P value** | **Q**  **value** | **VIP** | **AUC** |
| --- | --- | --- | --- | --- | --- | --- | --- | --- |
| EsomeprazoleNa | 346.12 | 4.18 | NA | NA | 0.04 | 1.00 | 3.34 | 0.74035 |
| Lenticin | 247.14 | 2.99 | HMDB0061115 | C09213 | <0.01 | 0.75 | 3.27 | 0.80702 |
| Indoleacrylic acid | 186.06 | 3.00 | HMDB0000734 | NA | <0.01 | 0.80 | 2.91 | 0.78246 |
| 2,2'-Methylenebis(4-methyl-6-tert-butylphenol) | 339.23 | 6.54 | HMDB0244434 | NA | 0.04 | 1.00 | 2.81 | 0.59649 |
| PC(20:4(5Z,8Z,11Z,14Z)/P-16:0) | 766.57 | 7.23 | HMDB0008455 | C00157 | <0.01 | 0.48 | 2.64 | 0.84561 |
| PC(16:0/9:0(CHO)) | 650.44 | 6.00 | NA | C00157 | <0.01 | 0.55 | 2.43 | 0.81754 |
| N-Methyl-2-pyridone-5-carboxamide (Nudifloramide) | 153.07 | 1.55 | NA | C05842 | 0.01 | 0.88 | 2.37 | 0.7614 |
| PC(20:4(8Z,11Z,14Z,17Z)/P-18:1(11Z)) | 792.59 | 7.22 | HMDB0008490 | C00157 | <0.01 | 0.75 | 2.16 | 0.81053 |
| SL 17:0;O/16:0 | 588.47 | 6.71 | NA | NA | 0.04 | 1.00 | 2.14 | 0.67719 |
| Lactosylceramide(18:1/16:0) | 862.63 | 6.85 | NA | NA | 0.01 | 0.90 | 2.07 | 0.72982 |
| Sulfur trioxide | 78.96 | 0.51 | HMDB0258610 | NA | 0.01 | 0.88 | 2.02 | 0.79649 |
| SM 26:2;2O/8:0 | 701.56 | 5.05 | NA | C00550 | 0.02 | 0.95 | 1.89 | 0.69474 |
| PC 22:2_18:5 | 832.59 | 8.54 | NA | C00157 | 0.01 | 0.88 | 1.85 | 0.74386 |
| PC(P-18:1(9Z)/18:3(6Z,9Z,12Z)) | 766.57 | 9.00 | HMDB0011311 | C00157 | <0.01 | 0.54 | 1.8 | 0.82807 |
| PC(20:2(11Z,14Z)/16:1(9Z)) | 784.58 | 8.54 | HMDB0008332 | C00157 | 0.01 | 0.90 | 1.73 | 0.75080 |
| 2-tetracosanamidoethanesulfonic acid | 474.36 | 6.24 | NA | NA | 0.03 | 1.00 | 1.66 | 0.68772 |
| Glyceraldehyde | 89.02 | 9.00 | HMDB0001051 | C02154 | 0.03 | 1.00 | 1.61 | 0.71930 |
| 2-amino-4-hydroxypyrimidine-5-carboxylic acid | 156.04 | 1.39 | NA | NA | 0.04 | 1.00 | 1.60 | 0.70175 |
| SM 32:2;2O/9:0 | 799.67 | 4.64 | NA | C00550 | 0.01 | 0.90 | 1.60 | 0.73333 |
| LPC O-18:1 | 566.38 | 5.19 | NA | NA | 0.01 | 0.90 | 1.49 | 0.71930 |
| Hexapropylene glycol | 367.27 | 3.62 | HMDB0341327 | NA | 0.04 | 1.00 | 1.47 | 0.69123 |
| PC(20:3(5Z,8Z,11Z)/P-18:1(11Z)) | 794.61 | 8.54 | HMDB0008392 | C00157 | 0.03 | 1.00 | 1.45 | 0.71228 |
| Docosahexaenoyl PAF C-16 | 792.59 | 8.49 | NA | NA | 0.02 | 0.95 | 1.45 | 0.74386 |
| LPC O-16:0 | 482.36 | 5.00 | NA | NA | 0.03 | 1.00 | 1.41 | 0.72281 |
| PS(20:0/22:6(4Z,7Z,10Z,13Z,16Z,19Z)) | 862.56 | 6.80 | HMDB0112537 | C02737 | 0.03 | 1.00 | 1.40 | 0.70175 |
| PC(P-18:1(9Z)/20:4(5Z,8Z,11Z,14Z)) | 792.59 | 8.96 | HMDB0011319 | C00157 | 0.02 | 0.95 | 1.37 | 0.72982 |
| 2,5-Di-tert-butylhydroquinone | 221.16 | 4.95 | HMDB0245492 | NA | <0.01 | 0.63 | 1.31 | 0.80351 |
| 5beta-Cholanic acid | 359.30 | 7.85 | HMDB0247018 | C19642 | 0.03 | 1.00 | 1.30 | 0.71228 |
| Ricinoleic acid | 297.25 | 5.49 | HMDB0034297 | C08365 | 0.04 | 1.00 | 1.17 | 0.69474 |

Abbreviations: MZ, Mass-to-Charge Ratio; RT, Retention Time; HMDB, Human Metabolome Database ID; KEGG, Kyoto Encyclopedia of Genes and Genomes ID; VIP, Variable Importance in Projection; AUC, Area Under the ROC Curve; P Value, Probability Value; Q Value, Adjusted p-value.

#

# Supplementary Table4. Spearman correlation coefficients between differential metabolites and clinical laboratory parameters.

| Metabolites | correlation coefficient/p-value | WBC(10^9/L) | RBC (10^12/L) | PLT(10^9/L) | NEU(10^9/L) | L(10^9/L) |
| --- | --- | --- | --- | --- | --- | --- |
| Hexapropylene glycol | r | -0.051 | 0.013 | -.226 | -0.021 | -0.099 |
|  | p-value | 0.776 | 0.94 | 0.198702 | 0.905 | 0.577 |
| 5beta-Cholanic acid | r | -0.012 | -0.122 | -0.118 | 0.08 | -0.323 |
|  | p-value | 0.945 | 0.493 | 0.505 | 0.652 | 0.062 |
| Ricinoleic acid | r | -0.01 | 0.031 | -0.23 | 0.053 | -0.217 |
|  | p-value | 0.955 | 0.862 | 0.191 | 0.766 | 0.219 |
| N-Methyl-2-pyridone-5-carboxamide (Nudifloramide) | r | 0.177 | 0.118 | 0.091 | 0.18 | -0.064 |
|  | p-value | 0.317 | 0.505 | 0.61 | 0.307 | 0.719 |
| PC(20:4(5Z,8Z,11Z,14Z)/P-16:0) | r | -0.396 | 0.186 | -0.34 | -0.449 | 0.112 |
|  | p-value | 0.021 | .293 | 0.049 | 0.008 | 0.53 |
| LPC O-16:0 | r | 0.202 | 0.281 | 0.033 | 0.102 | 0.23 |
|  | p-value | 0.252 | 0.107 | 0.854 | 0.567 | 0.191 |
| EsomeprazoleNa | r | 0.338 | 0.12 | -0.101 | 0.351^*^ | -0.123 |
|  | p-value | 0.051 | 0.498 | 0.569 | 0.042 | 0.487 |
| Docosahexaenoyl PAF C-16 | r | 0.04 | -0.006 | -0.013 | 0.067 | 0.158 |
|  | p-value | 0.822 | 0.973 | 0.94 | 0.706 | 0.373 |
| LPC O-18:1 | r | 0.116 | 0.103 | 0.109 | 0.11 | 0.078 |
|  | p-value | 0.515 | 0.562 | 0.539 | 0.537 | 0.661 |
| 2,5-Di-tert-butylhydroquinone | r | -0.374 | -0.179 | -0.174 | -0.28 | -0.212 |
|  | p-value | 0.029 | 0.31 | 0.324 | 0.109 | 0.229 |
| PC 22:2_18:5 | r | -0.066 | 0.061 | -0.073 | -0.076 | -0.1 |
|  | p-value | 0.712 | 0.734 | 0.683 | 0.668 | 0.957 |
| SL 17:0;O/16:0 | r | -0.289 | 0.072 | 0.049 | -0.29 | 0.096 |
|  | p-value | 0.097 | 0.688 | 0.784 | 0.097 | 0.588 |
| Glyceraldehyde | r | 0.179 | 0.159 | 0.149 | 0.109 | 0.044 |
|  | p-value | 0.312 | 0.368 | 0.399 | 0.54 | 0.804 |
| PC(20:4(8Z,11Z,14Z,17Z)/P-18:1(11Z)) | r | -0.45 | -0.101 | -0.401 | -0.374 | -0.121 |
|  | p-value | 0.008 | 0.568 | 0.019 | 0.03 | 0.497 |
| PC(16:0/9:0(CHO)) | r | 0.178 | -0.034 | 0.004 | 0.072 | 0.182 |
|  | p-value | 0.315 | 0.848 | 0.981 | 0.685 | 0.304 |
| PC(20:3(5Z,8Z,11Z)/P-18:1(11Z)) | r | -0.18 | 0.191 | 0.168 | -0.268 | 0.186 |
|  | p-value | 0.308 | 0.278 | 0.342 | 0.125 | 0.293 |
| 2-amino-4-hydroxypyrimidine-5-carboxylic acid | r | 0.22 | -0.161 | 0.051 | 0.221 | 0.029 |
|  | p-value | 0.211 | 0.363 | 0.776 | 0.21 | 0.869 |
| Lenticin | r | 0.21 | -0.07 | 0.04 | 0.15 | 0.05 |
|  | p-value | 0.234 | 0.695 | 0.82 | 0.398 | 0.781 |
| PC(20:2(11Z,14Z)/16:1(9Z)) | r | -0.108 | 0.06 | 0.249 | -0.96 | -0.16 |
|  | p-value | 0.541 | 0.737 | 0.156 | 0.59 | 0.928 |
| PS(20:0/22:6(4Z,7Z,10Z,13Z,16Z,19Z)) | r | -0.229 | -0.023 | -0.037 | -0.282 | 0.13 |
|  | p-value | 0.193 | 0.898 | 0.838 | 0.107 | 0.465 |
| SM 32:2;2O/9:0 | r | -0.404 | 0.006 | -0.116 | -0.409 | -0.188 |
|  | p-value | 0.018 | 0.973 | 0.514 | 0.016 | 0.287 |
| SM 26:2;2O/8:0 | r | -0.058 | -0.203 | -0.209 | -0.065 | -0.176 |
|  | p-value | 0.745 | 0.249 | 0.236 | 0.715 | 0.32 |
| PC(P-18:1(9Z)/18:3(6Z,9Z,12Z)) | r | -0.226 | -0.413 | -0.119 | -0.139 | -0.153 |
|  | p-value | 0.199 | 0.015 | 0.504 | 0.434 | 0.388 |
| PC(P-18:1(9Z)/20:4(5Z,8Z,11Z,14Z)) | r | -0.152 | -0.267 | 0.027 | -0.125 | 0.03 |
|  | p-value | 0.39 | 0.127 | 0.879 | 0.48 | 0.866 |

| Metabolites | correlation coefficient/p-value | M(10^9/L) | Glu（mmol/l) | LDL(mmol/L) | INR(s) | D-Dimer(mg/L) |
| --- | --- | --- | --- | --- | --- | --- |
| Hexapropylene glycol | r | 0.007 | -0.173 | -0.178 | -0.212 | -0.194 |
|  | p-value | 0.969 | 0.3292 | 0.315 | 0.236 | 0.270949 |
| 5beta-Cholanic acid | r | -0.094 | 0.125 | 0.003 | -0.248 | 0.044 |
|  | p-value | 0.598 | 0.48 | 0.988 | 0.158 | 0.804 |
| Ricinoleic acid | r | 0.083 | 0.006 | -0.067 | -0.032 | 0.03 |
|  | p-value | 0.639 | 0.972 | 0.708 | 0.859 | 0.867 |
| N-Methyl-2-pyridone-5-carboxamide (Nudifloramide) | r | 0.123 | 0.058 | -0.106 | -0.043 | 0.046 |
|  | p-value | 0.488 | 0.745 | 0.552 | 0.811 | 0.795 |
| PC(20:4(5Z,8Z,11Z,14Z)/P-16:0) | r | -0.439 | -0.111 | 0.073 | -0.377 | -0.044 |
|  | p-value | 0.009 | 0.532 | 0.681 | 0.028 | 0.804 |
| LPC O-16:0 | r | 0.172 | 0.029 | 0.119 | 0.097 | 0.044 |
|  | p-value | 0.33 | 0.871 | 0.501 | 0.584 | 0.805 |
| EsomeprazoleNa | r | 0.496^**^ | -0.049 | -0.063 | 0.132 | 0.284 |
|  | p-value | 0.003 | 0.783 | 0.722 | 0.458 | 0.104 |
| Docosahexaenoyl PAF C-16 | r | -0.031 | 0.034 | 0.009 | -0.053 | 0.008 |
|  | p-value | 0.863 | 0.848 | 0.959 | 0.765 | 0.962 |
| LPC O-18:1 | r | -0.001 | -0.103 | -0.005 | 0.201 | -0.019 |
|  | p-value | 0.994 | 0.562 | 0.979 | 0.255 | 0.913 |
| 2,5-Di-tert-butylhydroquinone | r | -0.235 | -0.097 | -0.177 | -0.265 | -0.219 |
|  | p-value | 0.182 | 0.587 | 0.317 | 0.131 | 0.213 |
| PC 22:2_18:5 | r | -0.218 | -0.156 | 0.03 | -0.058 | -0.277 |
|  | p-value | 0.216 | 0.379 | 0.867 | 0.745 | 0.112 |
| SL 17:0;O/16:0 | r | -0.333 | -0.29 | 0.27 | 0.101 | -0.244 |
|  | p-value | 0.054 | 0.096 | 0.123 | 0.57 | 0.164 |
| Glyceraldehyde | r | 0.225 | -0.104 | -0.019 | 0.174 | 0.189 |
|  | p-value | 0.201 | 0.56 | 0.913 | 0.326 | 0.285 |
| PC(20:4(8Z,11Z,14Z,17Z)/P-18:1(11Z)) | r | -0.251 | -0.097 | 0.118 | -0.354 | -0.086 |
|  | p-value | 0.153 | 0.587 | 0.506 | 0.04 | 0.627 |
| PC(16:0/9:0(CHO)) | r | 0.451** | 0.143 | 0.089 | 0.139 | 0.105 |
|  | p-value | 0.007 | 0.42 | 0.615 | 0.433 | 0.554 |
| PC(20:3(5Z,8Z,11Z)/P-18:1(11Z)) | r | -0.221 | -0.03 | 0.161 | -0.031 | -0.209 |
|  | p-value | 0.209 | 0.867 | 0.363 | 0.861 | 0.235 |
| 2-amino-4-hydroxypyrimidine-5-carboxylic acid | r | 0.12 | -0.009 | -0.332 | 0.004 | -0.029 |
|  | p-value | 0.499 | 0.958 | 0.055 | 0.983 | 0.869 |
| Lenticin | r | 0.16 | 0.518 | -0.109 | -0.063 | 0.13 |
|  | p-value | 0.367 | 0.002 | 0.54 | 0.725 | 0.463 |
| PC(20:2(11Z,14Z)/16:1(9Z)) | r | -0.06 | -0.243 | 0.059 | -0.054 | -0.254 |
|  | p-value | 0.736 | 0.166 | 0.739 | 0.76 | 0.147 |
| PS(20:0/22:6(4Z,7Z,10Z,13Z,16Z,19Z)) | r | -0.046 | -0.15 | 0.191 | 0.082 | -0.125 |
|  | p-value | 0.797 | 0.398 | 0.28 | 0.643 | 0.48 |
| SM 32:2;2O/9:0 | r | -0.194 | 0.088 | 0.189 | -0.108 | 0.05 |
|  | p-value | 0.272 | 0.621 | 0.284 | 0.543 | 0.78 |
| SM 26:2;2O/8:0 | r | -0.04 | -0.122 | 0.117 | -0.035 | 0.036 |
|  | p-value | 0.821 | 0.491 | 0.509 | 0.845 | 0.842 |
| PC(P-18:1(9Z)/18:3(6Z,9Z,12Z)) | r | -0.216 | -0.175 | 0.286 | -0.003 | 0.057 |
|  | p-value | 0.22 | 0.322 | 0.102 | 0.987 | 0.748 |
| PC(P-18:1(9Z)/20:4(5Z,8Z,11Z,14Z)) | r | -0.12 | 0.198 | -0.108 | -0.079 | -0.135 |
|  | p-value | 0.499 | 0.263 | 0.543 | 0.658 | 0.446 |

*Abbreviations:* WBC, white blood cells; RBC, red blood cells; PLT, platelets; NEU, neutrophils; LYM, lymphocytes; MON, monocytes; Glu, glucose; LDL, low-density lipoprotein; INR, international normalized ratio; D-dimer, D-dimer. Correlation coefficients (r) and p-values were calculated using Spearman's rank correlation test. Values in bold with asterisks indicate statistically significant correlations (* p < 0.05, ** p < 0.01).

**Supplementary Methods 1.** Experimental and analytical methods

Experimental process
1.1 Description of metabolite extraction

The collected samples were thawed on ice, and metabolite were extracted with 80% methanol Buffer. Briefly,100 μl of sample was extracted with 400μl of precooled methanol. The extraction mixture was then stored in 30 min at -20 ° C. After centrifugation at 20,000 g for 15 min, the supernatants were transferred into new tube to and vacuum dried. The samples were redissolved with 100 μL 80% methanol and stored at -80 ° C prior to the LC-MS analysis. In addition, pooled Q samples were also prepared by combining 10 μ L of each extraction mixture.

1.2Description of liquid phase parameters

All samples were acquired by the LC-MS system followed machine orders. Firstly, all chromatographic separations were performed using an UltiMate 3000 UPLC System (Thermo Fisher Scientific, Bremen, Germany). An ACQUITY UPLC T3 column (100mm*2.1mm,1.8µm,Waters,Milford,USA) was used for the reversed phase separation.The column oven was maintained at 40 ° C. The fter, 5mM ammonium acetate and 5mM acetic acid) and solvent B (Acetonitrile). low rate was 0.3 ml/min and the mobile phase consisted of solvent A . Gradient elution conditions were set as follows: 0～0.8 min, 2% B; 0.8～2.8 min, 2% to 70% B; 2.8～5.6 min, 70% to 90% B; 5.6~6.4 min, 90% to 100% B; 6.4~8.0 min, 100% B; 8.0～8.1 min, 100% to 2% B; 8.1～10 min, 2%B.

- 1. Description of mass spectrometry parameters

Q-Exactive：

A high-resolution tandem mass spectrometer Q-Exactive (Thermo Scientific) was used to detect metabolites eluted form the column. The Q-Exactive was operated in both positive and negative ion modes. Precursor spectra (70 -1050 m/z) were collected at 70,000 resolution to hit an AGC target of 3e6. The maximum inject time was set to 100 ms. A top 3 configuration to acquire data was set in DDA mode. Fragment spectra were collected at 17,500 resolution to hit an AGC target of 1e5 with a maximum inject time of 80 ms.In order to evaluate the stability of the LC-MS during the whole acquisition, a quality control sample (Pool of all samples) was acquired after every 10 samples.

2 Information analysis process

2.1 Information analysis description

The acquired MS data pretreatments including peak picking, peak grouping, retention time correction, second peak grouping, and annotation of isotopes and adducts was performed using XCMS software. LC−MS raw data files were converted into mzXML format and then processed by the XCMS, CAMERA and metaX toolbox implemented with the R software. Each ion was identified by combining retention time (RT) and m/z data. Intensities of each peaks were recorded and a three dimensional matrix containing arbitrarily assigned peak indices (retention time-m/z pairs), sample names (observations) and ion intensity information (variables) was generated.

The online KEGG, HMDB database was used to annotate the metabolites by matching the exact molecular mass data (m/z) of samples with those from database. If a mass difference between observed and the database value was less than 10 ppm, the metabolite would be annotated and the molecular formula of metabolites would further be identified and validated by the isotopic distribution measurements. We also used a in-house fragment spectrum library of metabolites to validate the metabolite identidification.

Statistical analysis was performed in R (version 4.0.0). The raw protein intensity will be

normalized by method "medium", Hierarchical clustering was performed using pheatmap package. Principal component analysis (PCA) was performed using metaX package. The PLSDA analysis is performed by the R package ropls and the VIP values of each variable are calculated.Correlation analysis was performed by Pearson correlation coefficient of cor package .The three conditions of P Value<0.05, difference multiple >1.2 obtained by T test and VIP calculated by PLSDA analysis simultaneously met the screening of the final metabolites with significant differences.

Hypergeometric-based enrichment analysis with KEGG Pathway was performed to annotate protein sequences. individually.The software GSEA (v4. 1.0) and MSigDB were used for gene set enrichment analysis to determine whether a set of genes in a specific KEGG pathway in different situations. Meeting this condition |NES|>1, NOM p-val<0.05, FDR q-val<0.25 were considered to be significantly different between the two groups. .The network map is drawn according to the pathway where the metabolite is located.

**
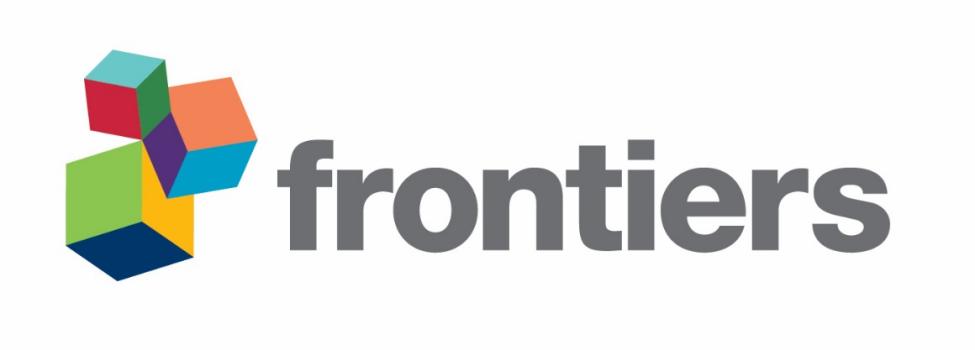
**
